# Hypergraph-Based Recognition Memory Model for Lifelong Experience

**DOI:** 10.1155/2014/354703

**Published:** 2014-10-13

**Authors:** Hyoungnyoun Kim, Ji-Hyung Park

**Affiliations:** Korea Institute of Science and Technology, Korea University of Science and Technology, Hwarang-ro 14-gil 5, Seongbuk-gu, Seoul 136-791, Republic of Korea

## Abstract

Cognitive agents are expected to interact with and adapt to a nonstationary dynamic environment. As an initial process of decision making in a real-world agent interaction, familiarity judgment leads the following processes for intelligence. Familiarity judgment includes knowing previously encoded data as well as completing original patterns from partial information, which are fundamental functions of recognition memory. Although previous computational memory models have attempted to reflect human behavioral properties on the recognition memory, they have been focused on static conditions without considering temporal changes in terms of lifelong learning. To provide temporal adaptability to an agent, in this paper, we suggest a computational model for recognition memory that enables lifelong learning. The proposed model is based on a hypergraph structure, and thus it allows a high-order relationship between contextual nodes and enables incremental learning. Through a simulated experiment, we investigate the optimal conditions of the memory model and validate the consistency of memory performance for lifelong learning.

## 1. Introduction

An advanced cognitive agent is required to contain abilities to interact with a nonstationary environment and to adapt to a changing situation by understanding the current situation through previously learned context. In terms of lifelong experience modeling, one of the essential properties of the cognitive agents is to incrementally update the new data without using previously learned information.

For a cognitive agent to update new data from its environment, the agent judges whether the input situation is known or unfamiliar compared with its previous experience and knowledge. If the situation is perceived as a previously faced event, the agent determines the successive procedure to either respond to the situation or associate other data related to the situation. In contrast, if it is confirmed that the current data are new, the agent selects the next procedure, such as learning the event or classifying it as a closely related event. This procedure generally works continuously according to the interactions during the lifecycle. In particular, the judgment process operates within the recognition memory.

In terms of lifelong learning, the cognitive agent requires both a memory model to encode the experienced data and a functional process to judge the input data through a comparison with the encoded memory. Psychologists and brain researchers have investigated the functional mechanism of judging input data through human experiments and anatomical evidence [1–4]. However, studies on a computational recognition memory model for lifelong learning remain insufficient. The previous research for computational models has been limited to static condition.

Lifelong experience has particular properties unlike other signal data. They are composed of various types of contextual attributes, which has a format of multivariate and categorical data. Each attribute has a relationship to other attributes including high-order relations. Considering the data property, in order to deal with the lifelong experience data, the memory model needs a flexible structure.

In this paper, we suggest a hypergraph-based memory model that enables contextual modeling and incremental learning. In order to build a computational recognition memory model for lifelong learning, we solve research issues of recognition memory in nonstationary environment. We show a human-like recognition performance via the proposed computational model based on content-addressable memory mechanism. In addition, the encoding and inference mechanisms of the proposed memory model are described, and the optimal conditions of the model obtained through empirical simulations are investigated. Through the simulated experiments, we show that the performance of the recognition memory model is similar to human and that the model is applicable to lifelong learning.

## 2. Recognition Memory

Recognition memory performs two functions, that is, knowing and remembering [5]. Knowing, also called familiarity, is about judging whether a single item has been previously experienced. Remembering is a process of recollection in which the associated items from an input are retrieved. Although there are controversial arguments regarding the structure and function of recognition memory, we developed a computational model for recognition memory based on the dual process theory [6–8], which differentiates these two functionalities.

Of these two functionalities, we focus particularly on familiarity as a fundamental step in implementing recognition memory. By considering the relation between familiarity and recollection, the controversial issues are mainly related to the functional position of recollection. We suggest a computational model based on a literature review, rather than proposing strong human behavioral evidence for a theory related to the role of recollection. Hence, in this paper, both the proposed model and experimental results are described based on the characteristics of familiarity.

### 2.1. Characteristics of Familiarity

Several models that define the properties of familiarity and recollection have been developed. In terms of familiarity, we summarize the properties from dual process theory models. Atkinson et al. described the signal detection theory (SDT) [9] in which the activation level between old and new items is controlled [10]. More specifically, Yonelinas et al. evaluated familiarity as a quantitative memory strength based on the SDT [11, 12]. They found that the activation level has a symmetric curvilinear shape in a receiver operating characteristic (ROC) graph when only familiarity operates without recollection. Mandler et al. and Jacoby et al. hypothesized that familiarity is highly related to implicit memory tasks such as word-stem completion [13–15]. Yonelinas et al. also argued that familiarity generally deals with two items that can be controlled both together and as single items [16–18].

It has also been debated whether the familiarity in recognition memory is related to both implicit and explicit memories [19, 20]. To satisfy the condition as explicit memory, familiarity is needed to show a regular ROC curve for the task of recognition judgment based on the SDT. The SDT supports the argument that input data with noise can be recognized as old data using similarity values. In contrast, the implicit property of recognition memory enables pattern completion such as word-stem completion. In terms of the SDT, a pattern completion task is construed such that input data with strong noise are converted to a complete data, and thus the input is recognized as old or new according to the completion status. Therefore, to construct a model for familiarity, both old/new judgment as an explicit memory and pattern completion as an implicit memory are considered simultaneously.

Another characteristic of familiarity is related to the ROC curve between true positive (hits) and false positive cases (false alarms). ROC curves for familiarity and recollection have different shapes from the viewpoint of the treated items. The performance of familiarity represents a symmetric curvilinear shape in the ROC curves [11]. Such a graph has been investigated through human behavioral experiments on recognizing words and images [21]. Although recognition certainty has been evaluated based on the subject's own feelings, many studies have shown similar results regardless of the data types and experimental setups. In particular, the shape of the ROC curves was shown to be constant regardless of the training sizes and intervals between training and recognition [8]. Both early encoded data and recently trained data contribute similarly to the performance of old/new judgments.

When we design and build a computational model for recognition memory suitable for lifelong learning, the properties of familiarity described above need to be considered. In the following, we survey previous studies on recognition memory at various computational levels.

### 2.2. Computational Models for Recognition Judgment

Recognition memory has been considered a special function of the human brain, rather than a structured type of memory. As a compositional model for the human brain, research into the cognitive architecture has tried to arrange special units for recognition memory. In ACT-R, list memory has an integrated structure that includes recognition memory [22, 23]. In this architecture, recognition memory is depicted as a simple function occurring in short-term memory and not in long-term memory. The model is unconcerned regarding the SDT or the difference between familiarity and recollection. Based on the ACT-R, a heuristic recognition test was executed for a simple binary judgment [24]. Soar, which is known to be a progressive architecture, judges familiarity according to the success of retrieval in episodic memory [25]. If the retrieval is successful, the input data are regarded as familiar. In this architecture, old/new judgment is not involved. This process considers the recollection process between two items as recognition memory.

In the above cognitive architectures, the recognition memory operates as an intelligent function working concurrently with implicit memory and association. However, an independent module for recognition memory is not involved. Recent research has tried to combine the recognition function on Soar. In particular, Li et al. proposed a mathematical approach to reduce the computational cost for searching through long-term memory [26]. This study contributed to the interactional functionality between the recognition memory and the existing cognitive architectures.

Mathematical and computational models for recognition memory have also been studied using the global matching algorithm. SAM [27], MINERVA2 [28], Matrix [29], and TODAM [30] are global matching models that judge familiarity by considering the relationship between a test item and memory [31]. In these models, judgment decision is made quickly, and the SDT is applied to evaluate the performance. The REM model judges the old and new using a Bayesian computation [32]. It computes a scalar value indicating the global matching between the test data and stored memory traces. Cox and Shiffrin advanced the issue of recognition memory by considering the dynamics [33]. According to the data type treated in recognition memory, the criteria for decision of judgment vary in acquiring a constant performance.

### 2.3. Our Approach

Previous computational models including cognitive architectures for implementing a cognitive agent have excluded the importance of incremental learning in memory. If we build a cognitive agent that works in a nonstationary dynamic environment, the agent needs to consider the ability to encode and update everyday experiences and recall or expect the exact data from memory. Moreover, lifelong experiences are composed of various types of contextual information and activities. Each attribute has a special relationship to other attributes. Sometimes, more than three attributes are synchronized to composite a situation. Therefore, we consider that the experience data need to be modeled in a temporal relationship between input events and even in a causal relationship between contextual values.

From the previous theories on familiarity, a computational model for recognition memory is required to satisfy the human-like performance of old/new judgments. So far, however, the exact mechanism of human brain is not fully investigated. The relationship between familiarity and recollection is still controversial. Some important issues of recognition memory such as aging, forgetting, and context dependency are unsolved yet. To approach the human-like cognitive model, the model may follow a neural mechanism like human or it may show a similar performance to human. In this paper, we try to show a similar performance of familiarity judgment while dealing with lifelong experience data to evaluate the model. If the suggested model reveals a human-like performance by comparing the ROC curves, we can further investigate the undisclosed characteristics of recognition memory from the memory model.

To enable lifelong learning in recognition memory, we apply a flexible structure to implement the computational memory model. Particularly for the role of familiarity judgment in recognition memory, we suggest a computational memory model that enables lifelong learning by applying a hypergraph structure. The model is built based on the concept of content-addressable memory. It records the data into the model without filtering or modifying. The hypergraph also supports a high-order relationship between nodes. By building a layered hypergraph structure, temporal events can be integrated into a network.

Through the memory model, in this paper, we try to answer the following questions.

- Does the proposed memory model show a human-like ROC performance of familiarity judgment?
- What is the remarkable characteristic of the proposed recognition memory model for treating lifelong experience data?
- Does the memory model maintain the performance of both familiarity judgment and pattern completion under nonstationary encoding conditions?

In the following sections, we introduce the hypergraph-based memory mechanism and evaluate the memory model, which shows a similar performance to the human tasks of recognition judgment. Furthermore, in terms of lifelong learning, we investigate the characteristics of the proposed recognition memory model under various hypergraph configurations. By considering temporal properties including the study duration, we validate the consistency of the performance. In addition, the memory model is compared with conventional probabilistic model to evaluate the performance of expectation in nonstationary environment.

## 3. Hypergraph-Based Memory Model

We propose a hypergraph-based memory model that enables incrementally encoding nonstationary contextual data and operating recognition judgment from the encoded memory model. In this section, we describe the memory mechanism, including encoding and judgment, from the concept of a hypergraph structure. The basic concept of the memory model follows the principles of a cognitive agent suggested by Zhang [34]. The hypergraph structure mimics brain mechanism related to memory encoding and retrieving. For memory encoding, input data are disassembled into subsets and distributed for storage in memory. To retrieve the data, segmented subsets are composited to generate the complete data. The primary processes of memory encoding and judgment from the memory are partitioning and combining. To support these memory mechanisms based on a subset combination, we apply a hypergraph structure and modify the structure by constructing a layered hypergraphs.

### 3.1. Hypergraph-Based Memory Structure

**Figure 1:**
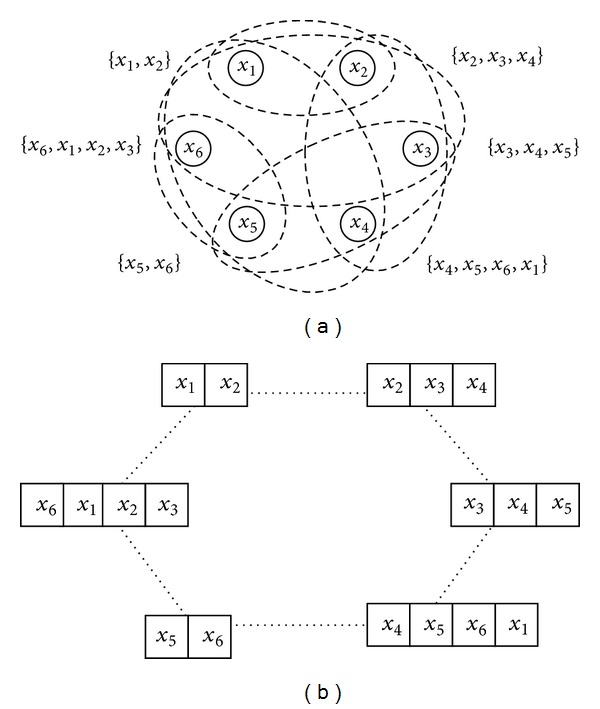
Graphical diagram of a hypergraph-based structure. (a) A hypergraph with six nodes and six edges. (b) A hypergraph structure constructs circular connections inside the network when the data comes from contextual events.

A hypergraph is a graphical model composed of edges, which are combinations of nodes [35]. When an event instance *X* is {*x*_1_, *x*_2_,…, *x*_6_}, a hypergraph can be represented as shown in Figure 1(a). In a hypergraph, a complete instance is divided into several subsets, which share a common property. Each node is allowed to be included in distinguished subsets according to the endowed parameter conditions. A single subset, combination of nodes, is assigned as a hyperedge with *k* nodes, where *k* is a variable indicating the size of the nodes in a subset.

The structure of a hypergraph has the advantage of building high-order relationships. Using the flexible combinatorial structure of a hypergraph, several research domains have applied such characteristics as a spatial relationship in image processing and a temporal relationship in formal language analysis [36–38]. A hypergraph structure is adaptable to build relations of contextual data and serial data.

**Figure 2:**
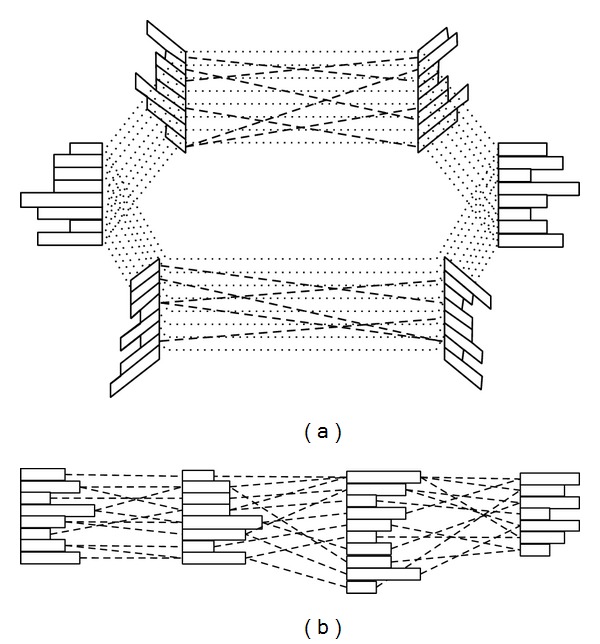
Hypernetwork structure generated by accumulating hypergraphs. The solid rectangles indicate edges with different node sizes. The dotted lines indicate the links between two edges acquired from the input data. According to the property of instances, a hypernetwork is shaped like a ring (a) or a line (b).

To make a dense connection inside the data, a hyperedge includes* links* with each weight between adjacent edges such that the hyperedges are fully connected. For example, if a hypergraph tries to model contextual event instances which are composed of six attributes, each edge is composed of *k* nodes including the node in the order of dimensions. In this case, the hypergraph structure is modified into the shape of a circular network, as shown in Figure 1(b). The connection of links is dependent on the characteristic of encoding data. The contextual data composed of categorical values has no prior order between nodes. In comparison, if words are encoded into a hypergraph structure, each letter has a serial order so that the link connection has a linear network (see Figure 2(b)).

Inside the hyperedges, links with weights are created. In terms of nodes, an edge structure represents a strong relationship between nodes in the edge. On the other hand, a link structure indicates a weak relationship. Therefore, a single node comes to have various relations with the whole event instance. This means that a high-order relationship can be accomplished according to the circular or linear configuration of the edges and links.

A hypergraph structure is suitable for modeling nonstationary contextual relationships. A hypergraph allows incremental learning by accumulating other hypergraphs into the previous structure. When an event instance is entered, it is replaced with a hypergraph. If other event instances with the same dimensional properties are entered, that is, the same attributes with different values, hyperedges can be shared to represent their hypergraph. Temporal event instances are accumulated in a hypergraph structure. Hyperedges have various links with adjacent hyperedges based on the input instance. The layered hypergraphs become a network, which we therefore call a* hypernetwork*.  Figure 2 shows the shape of a hypernetwork. The network shape is determined according to the dimensions of the instances and the configuration of the hyperedges as well as the property of instances.

The proposed hypernetwork enables incremental learning. Edges from a hypergraph can be accumulated into a hypernetwork according to alignment of their structure. It needs not previously encoded event data. To update the hypernetwork, an input instance goes through sampling, connecting, and weighting steps. At first, an instance is sampled into hyperedges with order *k*. After investigating the duplications between the new hyperedges and the edges in the memory, the matched or created edges are connected with each other. A number of connections is accumulated such that the weight of each connection changes.

A higher count indicates a strong relationship. The accumulated number of connections between two hyperedges is represented as a positive number. To emphasize the initial connection between two associated edges and to normalize the weights, the weight of links forms a half sigmoid function. The maximum value of the weight is given to 1.0. The graph for the weight follows a monotonous slope. Equation (1) represents the sigmoid function for weights:

(1) $$\begin{array}{c} \varphi_{ij}=f\left\{{\left(1+\exp\left(-\frac{l_{ij}}{C}\right)\right)}^{-1}\right\}. \end{array}$$

Here, *φ*_*ij*_ indicates the link weight between hyperedges *i* and *j* from different edge sets, *l*_*ij*_ is the accumulated number of connections, and *C* is a constant for modulating the slope of the sigmoid function. A larger *C* slows the grade, where *C* is empirically determined to avoid early convergence.

#### 3.1.1. Model Parameters

The hypernetwork has a data-driven structure. From the input data, the fixed dimensions of the data determine the structure of a network. Inside the network, several edge configurations can be applied. Edge configuration depends on both the order size of edges *k* and the combination of edge types. Outside the network, the learning procedure, such as the number of repeated encodings, can be modulated.

To modulate the structure, the hyperedge configuration is essential. Generally, a *k*-hypergraph is composed of *k* uniform hyperedges, where the length of the hyperedges is assigned as *k*. If *k* is fixed, we find an optimal configuration by modulating the magnitude of *k*. If *k* is variable, a hypernetwork is built with the mixed properties of different order sizes.

Another parameter of the hyperedges is the combinational type used to compose a hyperedge. One hyperedge includes the serially adjacent nodes in the data. However, the serial order of the data does not assure a close relationship among the data attributes. Furthermore, when knowledge of the causal relation of the attributes is absent, the serial order will influence the encoded model inadequately. Hence, a way to combine edges from the attributes is important for building a memory model with high-order relationships.

The last parameter that affects the structure of a hypernetwork is the repetition of data encoding into the memory. After an instance is encoded once, what happens if the instance is encoded again? Repeated encodings are interpreted as the study duration in recognition memory [39–41]. For a single instance, multiple encodings can affect the performance and structure of the memory model. According to the durational study, a hypernetwork can be a dense or coarse network. Consequently, the parameters that influence the memory structure are the relation between attributes, the size of edge order, the combinational order of the edges, and the repetition of the encoding and retrieval.

#### 3.1.2. Scalability

The proposed hypernetwork stacks input data into memory as the data accumulates. For lifelong experience, the length of the incoming data is temporally unlimited. Thus, our concern regarding the memory model is the capacity of the patterns covered. The main characteristic of the memory structure is reflecting on the partitioning and combining of the data.

When we define the number of values of each contextual attribute as *C*_*i*_, where *i* ranges from 1 to *d* (dimension of attributes), the possible combinations of instances are ∏_*i*=1_^*d*^ *C*_*i*_. If we set the fixed order size, *k*, the possible combinations of edges are represented as follows:

(2) $$\begin{array}{c} \overset{k}{\underset{i=1}{\prod }}C_{i}+\overset{k+1}{\underset{i=2}{\prod }}C_{i}+\cdots +\overset{k+d-1}{\underset{i=d}{\prod }}C_{i}=\overset{d}{\underset{t=1}{\sum }}\left(\overset{k+t-1}{\underset{i=t}{\prod }}C_{i}\right). \end{array}$$

If we assume *C*_1_ = *C*_2_ = ⋯ = *C*_*d*_ = *C*, the ratio of possible edges over possible instances is *dC* ^*k*−*d*^. Usually, *k* is smaller than *d* and *C* is larger than *d*. Accordingly, *dC* ^*k*−*d*^ is less than 1, and thus the numbers of created edges and links can more quickly converge than those of the instances. By using smaller number of edges, our proposed hypergraph structure can represent the entire instance combinations.

### 3.2. Inference Mechanism

In summary, the proposed memory model is a layered hypergraph-based network. To operate as a recognition memory model, the model needs to facilitate both familiarity judgment and pattern completion. In this section, we deal with the judgment mechanism of hypergraph-based memory.

In terms of the memory mechanism, there are two types of memory, activation-based and weight-based memory mechanisms [42]. A weight-based mechanism uses the weights in the networks. A summation of all related weights is used to judge the classification of the input instance and categorize the output [43]. Previous global matching algorithms were built on the weight-based mechanism [31]. On the other hand, an activation-based mechanism adopts the shape of activation patterns as a judgment criterion. Previous researches on memory models have approached the functionality using a distinctive mechanism rather than mixing these two different mechanisms together [42, 44]. However, a hypernetwork has a particular connectivity in its structure and an individual weight for each connection, and thus the model represents two memory mechanisms together. As an activation-based mechanism, the model uses the shape of the activated edges and their connections. A weight-based mechanism enables measuring the intensity of the connections using the link weights. From the encoded memory, we describe the judgment mechanism of recognition memory through the two memory mechanisms.

#### 3.2.1. Familiarity Judgment

**Figure 3:**
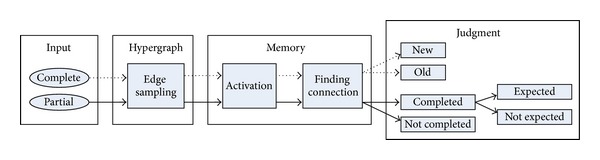
The recognition judgment procedure according to the type of input data. The upper arrows (dot lines) represent a process of familiarity judgment from complete input data. In contrast, the lower arrows (solid lines) show pattern completion from partial input data.

A constructed memory encodes all data into a hypergraph structure. When the new input data enter the memory, a recognition judgment begins. According to the completeness of the input data, the process for the judgment is separated (see Figure 3). When an input has no missing value, the result of the judgment is whether the input is old or new. On the other hand, a partial input to be judged requires distinctive processes related to the pattern completion. Inside the memory, the data commonly pass through the steps of edge sampling, activation, and finding fully activated connection.

The recognition judgment mechanism is divided into two steps: activation and judgment. The first, activation, is a step for finding the matched hyperedges from the input data. For an input probe, only few edges are matched and activated. The memory model infers the recognition from these small portions of the entire memory. When input data are observed, edges based on the data are extracted with regard to the edge configuration of the model. In the activation step, the extracted edge, *E*_*i*_, and previously encoded edge, *E*_*m*_, are compared.

To check the correspondence between two edges, the inclusion relation is applied for a comparison measure. As a condition of the activation between two edges, at least one value should be matched, and no mismatched values should exist. The activation function can be represented as follows:

(3) $$\begin{array}{c} \delta \left(E_{i},E_{m}\right)=\{\begin{array}{cc} 1, & \text{if}\;(\#\;\text{of}\;\text{matched}\;\text{value}>\text{Nm},\\ & \;\#\;\text{of}\;\text{mismatched}\;\text{value}=0)\\ 0, & \text{otherwise}. \end{array} \end{array}$$

**Figure 4:**
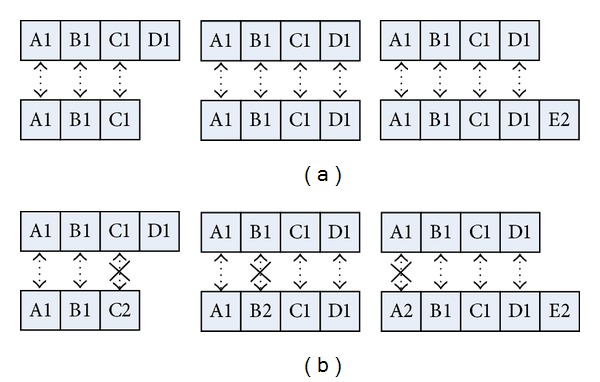
Conditions of edge activation: (a) successful and (b) failed activations. The top and bottom rows are for the input edges and encoded memory edges, respectively. Arrows with a cross indicate mismatches of the edges between the input and memory.

If one value of an edge is missing because of different edge lengths, we do not count this case as a mismatched value.  Figure 4 shows the success and failure of activation.

The secondary step of the recognition mechanism is judgment. To judge the familiarity, an activation-based memory mechanism is involved. The model investigates whether the activated edges construct a fully connected links. After the edges are selected in the memory, the connected links are activated consecutively. If two adjacent edges are activated simultaneously by the input, the connected link is finally assigned as an activated link. If edges are activated and connected with each other in every dimension of the network, the input data are judged as old (see (4)):

(4) $$\begin{array}{c} \underset{i}{\prod }\delta \left(E_{i},E_{m}\right)=1. \end{array}$$

**Figure 5:**
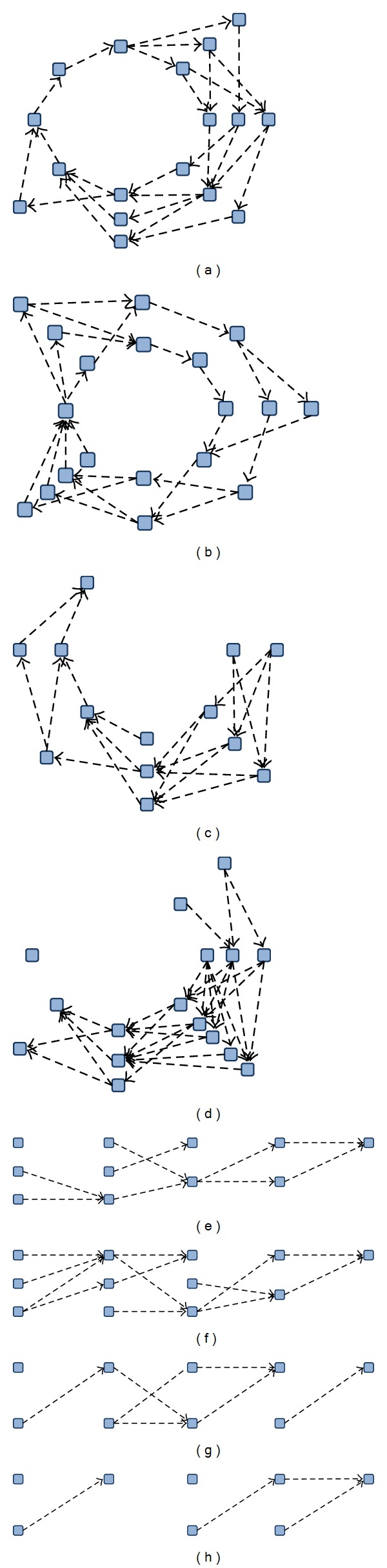
Network diagrams built using activated edges and links. Top graphs ((a)–(d)) represent ring-type networks and bottom graphs ((e)–(h)) represent line-type networks. Among the graphs, (a), (b), (e), and (f) contain a fully connected links, which are judged as old or familiar. On the other hand, other graphs ((c), (d), (g), and (h)) are judged as new or unfamiliar.

If the activated edges are fully connected in the memory network, it means that the combination of edges was previously encoded. The reason for this is that all of the encoded instances make a closed link set in the network model.  Figure 5 shows ring-type and line-type networks that have been judged as old or new. As shown in Figure 5, different edges are activated simultaneously. The number of closed loops changes according to the input data and network connectivity. However, the number of loops does not indicate certainty of the recognition judgment. The criterion is whether a fully connected link exists or not.

#### 3.2.2. Performance Measure

As a performance measure, we use a confusion matrix. In a hypernetwork, an old input is always judged as old if we assume that there is no removal of edges or links in the memory. This means that a false negative does not occur. Likewise, results judged as new are constantly made from new inputs. Our concern is false-positive cases, where a new input is judged as old. The subsampled structure allows various combinations of edges and thus the high connectivity enlarges the number of false positive cases. The main problem here is related to the connectivity. In the model, the connectivity indicates the complexity of network, which is the ratio of the average number of links per edge. According to the edge configurations, the connectivity varies, and the scale of the structure then changes along with the performance as recognition memory. Hence, to make an optimal structure for particular data, we need a rule to determine whether the performance in a certain configuration is suitable.

As above described, the activation-based memory mechanism provides a fast judgment on familiarity. However, there is a binary result of judgment: old and new. To measure the certainty of recognition of the input data, we additionally apply the weight-based mechanism. Each link between two edges has a weight. The activated edges and corresponding links are extracted from the input data. If there is a closed link connection, the summation of weights for activated links is assigned as a similarity using (5) as follows:

(5) $$\begin{array}{c} S=\underset{i,j}{\sum }\varphi_{ij}. \end{array}$$

We next consider two additional cases for calculating a similarity. For example, an open connection between activated edges excludes weights of inactivated dimension (see Figures 5(c), 5(d), 5(g), and 5(h)). In terms of activation-based mechanism, the memory judges the shape as being new. Regardless of the judgment, the input data elicit the similarity by giving a weight of zero. Another consideration is related to multiple extractions of closed connections. All extracted routes have their own similarity. The maximum similarity is selected for the input data.

The calculated similarity is used to draw ROC (receiver operating characteristic) curves. Even though the proposed mechanism judges the familiarity based on the activation patterns, a similarity further provides a precise performance based on the certainty of the input data from the ROC curve. The result may be evidence validating whether the proposed memory satisfies the human performance of recognition tasks. We investigate the optimal configuration of hypergraphs to resemble a human-like recognition memory model in Section 4.

#### 3.2.3. Pattern Completion

Another function of recognition memory is completing data from a partial input data. The proposed memory model allows the same functionality along with a judgment mechanism. The discriminative aspect compared with a recognition judgment is related to both the activation rule and the judgment rule. Unlike a familiarity judgment, input data for a pattern completion contains missing values. Extracted edges from the input also include the missing part. From the activation rule, two conditions are considered (see (3)). First, no mismatched value is allowed. Second, the number of matched values between two edges should be more than 0. Some edges involving missing values do not guarantee matched values even if there is no mismatched value.

Inference from partial data aims to generate a missing part using previously encoded memory. According to the memory structure, the generated data are recognized as familiar. Hence, the generation process involves reconstructing the missing data and extracting the complete data. The activated edges in the memory from partial input data build a full connection in the network, which represent completed data. After completion, the performance is estimated in two ways. One is the status of completeness, that is, whether the memory finds a full connection. The other is an expectation of whether one of the completed data points reconstructs the original data point exposing missing values. Similar to a familiarity judgment, the configuration of the hyperedge influences the performance of both completeness and expectation. A high connectivity to the memory has the potential to create a high completeness and expectation performance.

## 4. Experiment

A hypergraph-based recognition memory model was designed to build a recognition memory in lifelong experience. According to the data of experience, a distinguished type of hypernetworks is constructed. If we consider human activities in lifelong learning, our experiments can be set up to evaluate the performance of incremental learning for contextual data. In the experiment, we search the optimal edge configuration of the proposed memory model to resemble human performance on familiarity judgment. Then, we evaluate the performance of both old/new judgment and pattern completion in a nonstationary environment.

### 4.1. Experimental Design

In order to evaluate the model, we applied the* Reality Mining* dataset, which is composed of categorical and multivariate phone usage logs [45, 46]. We reorganized the Reality Mining data to contain eight attributes having contextual information and phone usages.  Table 1 shows the included attributes and their values. A total of 106 subjects participated in the dataset, and the logs were recorded automatically using the cell phones provided. In our experiments, the logs were converted into a sequential event stream with eight dimensions. According to the subjects, the number of events accumulated over a 9-month period reached around 7,000. For the experiment related to lifelong learning, we selected several subjects with large event instances.

The serial event streams were encoded one by one. Since the hypernetworks enable incremental learning, the model is able to update new incoming event data on the previously encoded hypernetworks without relearning. To investigate the performance of the recognition memory related to familiarity, the input data were divided into two types: complete and partial data. As shown in Figure 3, for complete data, the judgment is whether the input data are old or new. On the other hand, partial data go through the same procedure as complete data but the judgment considers whether partial data can be completed to the original data.

**Figure 6:**
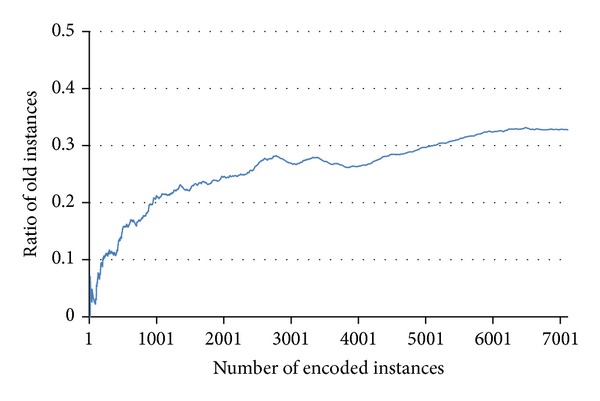
The ratio of old instances among total encoded instances during incremental learning. The overall ratio of old instance is about 32.8%.

In the Reality Mining data, each instance has eight attributes and the values change according to logging time. The combination of eight attributes composes an individual instance. If a new instance is equal to one of the previous events, the instance is regarded as old. Otherwise it is assigned as new. For the whole data, the ratio of old events changes and is represented in Figure 6. As shown in Figure 6, the ratio of old events gradually increases up to 32.8%. Kim and Park found the regularity in human behaviors from Reality Mining data [46]. In lifelong experience, we postulated that the human behaviors are repeated so that old/new judgment from the event stream is an important task to determine the next process such as updating the model or expecting the next situation.

**Figure 7:**
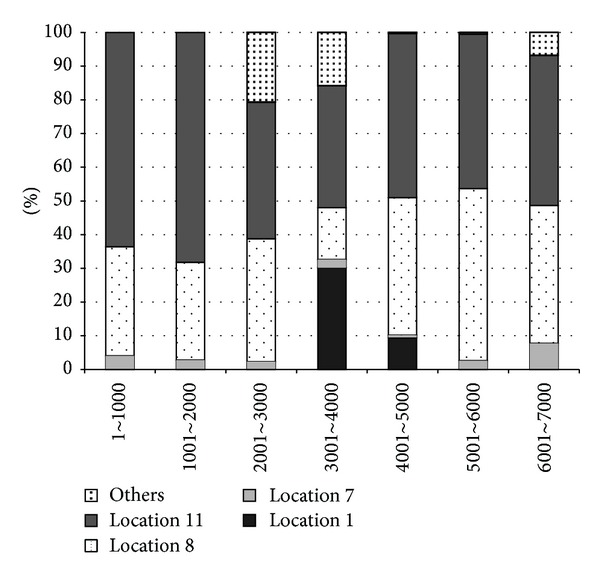
The distribution of an attributes, location, among eight attributes in Reality Mining dataset. The distribution changes according to the logging time.

We also found that the distribution of attributes changes by time. When we divide the whole data into seven sections with the same instances, each section has different distributions of attributes.  Figure 7 shows a change of distribution for one of attributes, location. Among over 30 values for location, four specific locations are dominant in the distribution. However, the distribution is changes by the logging time. If the attributes are modeled by probabilistic approach, each section needs a particular probability distribution table. Therefore, in human behavior modeling, we need to consider both the regularity of the overall event stream and the irregularity of local fluctuation inside the attribute.

The primary goal of the experiment is to evaluate the proposed memory model that represents the properties of human-like recognition memory. Whenever the Reality Mining data are encoded, the results of the recognition judgment were compared with human behavioral performance. In addition, the dataset contains contextual information so that when a partial data with missing attributes appears, the recognition memory completes the missing part and expects the next context from the previous experience. In the following experiments, we investigate the structural configuration of the proposed memory model to reveal the most similar human performance. Furthermore, we figure out the characteristics of the model in nonstationary environment and evaluate the performance of expectation in comparison with conventional probabilistic model, Bayesian networks.

### 4.2. Experiment 1: Find Optimal Edge Configuration

In the first experiment, we find the optimal hyperedge condition to derive acceptable results for judgment. Based on the hypergraph theory, the experimental dataset could be constructed into various hyperedge structures. Since the number of attribute is fixed as eight and each attribute can have a distinctive relationship, we apply a ring-type hypergraph structure. We classified three types of edge configurations. The first edge has a fixed order size within the range of 2 to *d* − 1, where *d* is the number of dimension. The second configuration sets random order *k*, which is determined to be between *r*_1_ and *r*_2_, where *r*_1_ and *r*_2_ are in the range of 2 to *d* − 1. In the above two edge configurations, the node combination is sequential in order of the number of attributes. Under the assumption of a random edge order, the third type of edge randomly selects order combinations. We applied a total of 13 edge categories, which include fixed-order edges, random-order edges, and random-edge combinations.

**Figure 8:**
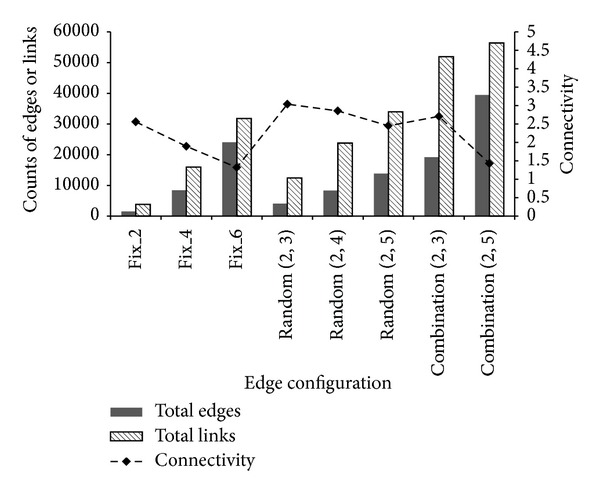
Scale of encoded memory and the memory connectivity according to the edge configuration.

Next, we investigated the incremental trend of recognition memory according to the amount of encoded data. Eight categories of edge configurations were compared.  Figure 8 shows comparison of the encoded memories in terms of the number of edges and links. Additionally, the ratio of links over edges, which is defined as connectivity, indicates the degree of memory density. For a recognition judgment, we expected the connectivity to play a critical role. When fixed-order edges are applied, the scale of the encoded memory increases, and the connectivity decreases. Both random-order edges and random-edge combination also showed this same tendency. However, the average connectivity achieved the maximum value in the case of random-order edges.

#### 4.2.1. Familiarity Judgment

In the experiments, we were mainly concerned with finding the evidence indicating that the proposed model has a similar recognition judgment performance as a human being. The Reality Mining dataset is appropriate to represent human behavior because it contains repeated similar events and changes the data distribution by time. We know that an optimal ROC curve has a high hit rate and low false alarm rate. However, human behavior has uncertainty with false alarms and false negatives. According to the various edge configurations, in this experiment, we drew ROC curves and investigated their properties to search for a human-like configuration. To draw a ROC curve, a similarity measure was applied. Even when a familiarity judgment is executed using the activation-based mechanism, we can acquire a similarity of the activated link using the weight-based mechanism. When the recognition memory uses only the activation-based mechanism, an input instance with a high similarity value can be judged as new. On the other hand, an input with a low similarity value is judged as old if all of the activated edges are connected with each other. For a familiarity judgment, we ignore this situation because we need to obtain quantified data for the ROC curve.

**Figure 9:**
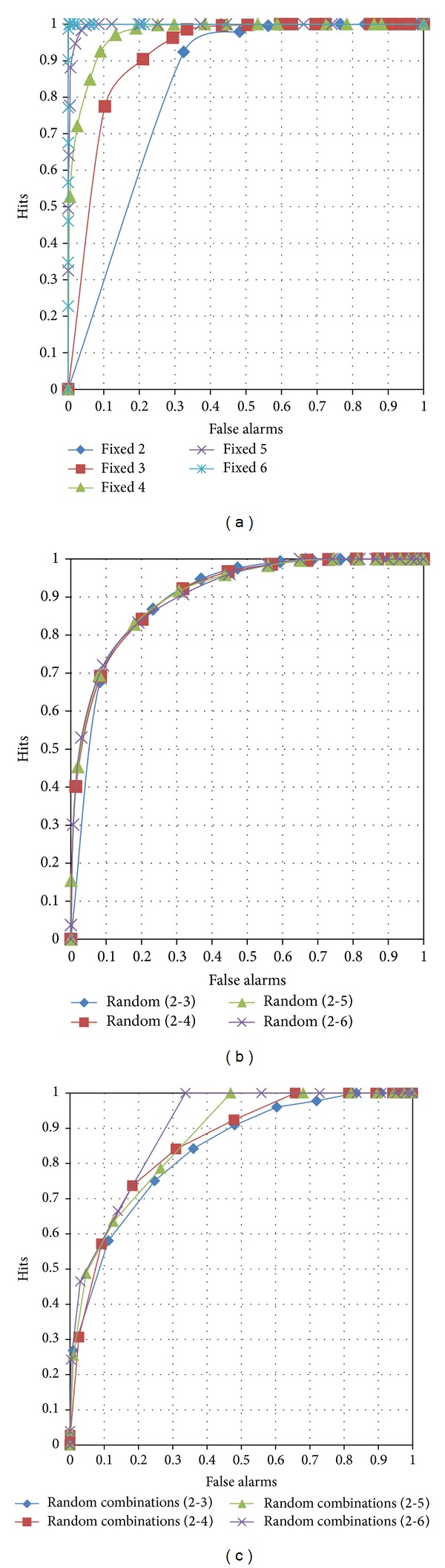
ROC curves for familiarity judgment according to the edge configuration: (a) fixed-order edges, (b) random-order edges, and (c) random-order edges with random combinations.

ROC curves for the three edge configurations with an order range between 2 and 6 are shown in Figure 9. For a fixed order of edges (see Figure 9(a)), higher-order edges derive more precise judgment. If the order size is over 5, the judgment is perfect. Lower-order edges have asymmetric ROC curves, unlike in human beings. Fixed-order edges do not guarantee regular ROC shapes despite the order size. However, when the edges are composed of various edge sizes, we can see curved symmetric ROC graphs, as shown in Figure 9(b). The order composition differs from 2 to 6, and the curves appear to be regular. High-order edges increase the hit rate, and low-order edges affect the false alarms. The point of interest here is that the curves were regular even though the false positive cases were changed according to the range of random orders. The third edge configuration contains randomly combined edge orders. The overall performance is better than the second edge configuration (see Figure 9(c)). The hit rate converges early when the edge order range is larger. However, like the fixed order edges, the curves show asymmetric shapes. Through the judgment experiment, we validated that a random edge configuration is most adaptable to the human-like recognition memory model.

#### 4.2.2. Pattern Completion

Another functionality of familiarity in recognition memory is pattern completion. From the connectivity graph, we can see an inversely proportional relation between familiarity judgment and network connectivity. A high connectivity between edges hinders the memory from judging new instances. Likewise, the property of network connectivity also influences the performance of pattern completion. We predicted that the number of activated edges and links enables a whole instance to be completed from partial input data.

Among the eight attributes in the Reality Mining data, we randomly selected three attributes to assign missing values in the input data. We then tested whether the memory generates the missing values. Furthermore, we evaluated whether the generated values are identical to the original input data. The former result was assigned as the completeness rate and the latter was the expectation. We drew the change in performance for both the completeness and expectation according to the edge configuration.

**Figure 10:**
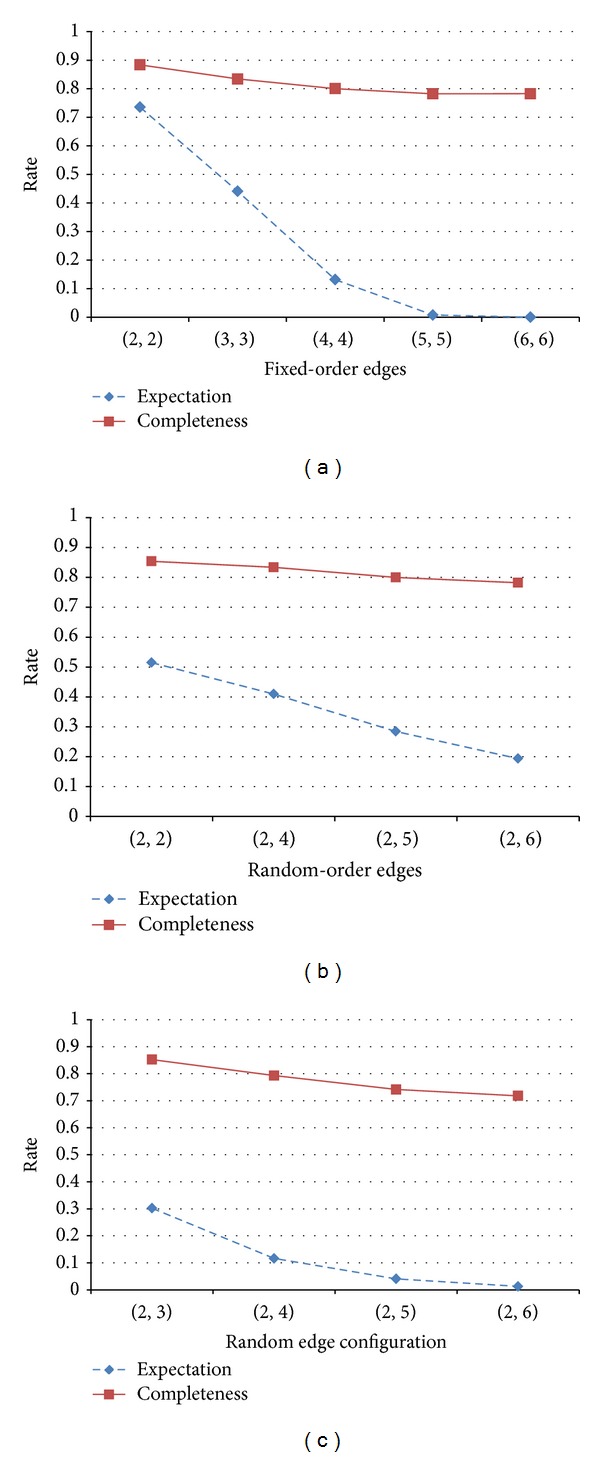
Performance of pattern completion according to the edge configuration: (a) fixed-order edges, (b) random-order edges, and (c) random-order edges with random combinations.

Figure 10 shows the pattern completion performance. Similar to our assumption, the overall performance was aided by the network connectivity. In case of two fixed-order edges, the completeness and expectation rates were the highest. However, the performance decreased drastically as the order size increased. Random-order edges with a random combination also showed a similar trend. The network connectivity directly affected the performance. When the memory was composed of random-order edges, the pattern completion performance slowly changed according to the change in connectivity.

From these two experiments, we evaluated the optimal human-like edge configuration for both a familiarity judgment and pattern completion. For these two tasks, fixed-order edges showed a trade-off with the order sizes. Random-order edges with a random combination also showed a similar trend as fixed-order edges. In comparison, random-order edges showed regular ROC curves and a reasonable pattern completion performance regardless of the range of random orders. Hence, in the next experiment, we investigated the temporal properties of the proposed recognition memory model based on a random-order edge configuration.

### 4.3. Experiment 2: Investigate Temporal Encoding

For the second experiment, we considered the properties of lifelong learning. We investigated the memory model in terms of the study duration and scale of encoded memory. Based on the first experiment, we evaluated whether the proposed recognition memory model resembles human performance through a comparison of the ROC curves. Later in the experiment, we investigated whether the scale of memory affects the familiarity judgment performance. Human familiarity capability was expected to be consistent regardless of the scale of information. However, previous computational models on recognition memory have ignored this condition. Hence, we proved that our model is superior for lifelong learning by showing the performance consistency at different scales of encoded memory.

#### 4.3.1. Temporal Encoding with Different Scale of Memory

As the edge configuration, we assigned random-order edges with a range of (2, 5), which include as many various edge orders as possible. The dataset has about 7,000 instances in temporal order. In the same manner of evaluation, an instance is judged as old or new before the input instance is encoded. The performance was recalculated for every 1,000 instances that were encoded. The dataset was divided into seven subdataset. In the first subdataset, there are no previously encoded data. In the second subdataset, 1,000 instances are tested in memory where previous 1,000 instances were encoded. In the seventh subset, 6,000 instances have been encoded into memory, and the remaining instances are judged for evaluating the ROC curve.

**Figure 11:**
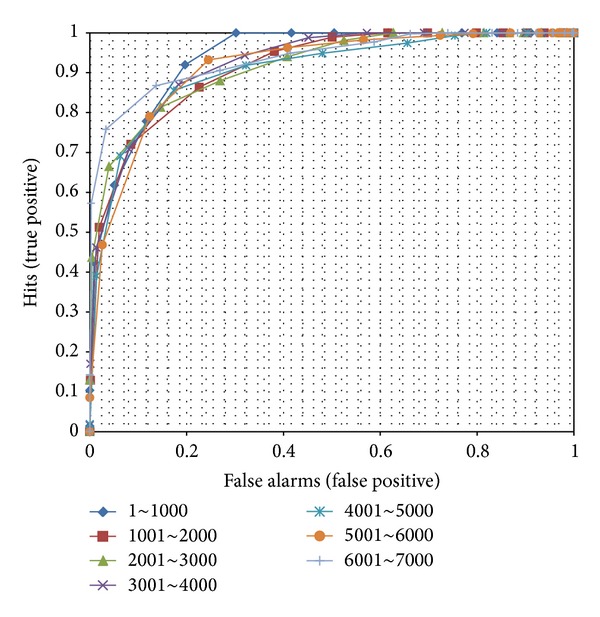
ROC curves with different scales of memory. Each curve is calculated using different scales of encoded memories and the same number of data to judge familiarity.

Figure 11 shows the ROC curves with different scales of memory. Overall, the shapes of the curves are constant except for the first and last sections. In the first section, the judgment performance was the highest. In contrast, the last section showed the lowest performance. However, the other middle sections were indistinguishable. Our proposed model produces a rather regular trend for temporal encoding.

#### 4.3.2. Study Duration

Another property of lifelong learning is related to study duration. The study duration reveals how the repeated encodings and observations influence the memory performance. The assumption of the study duration is related to the hyperedge configuration. If a single event instance is regularly subsampled into a certain number of hyperedges with a fixed order, repeated samplings can be ignored. Otherwise, in a random hyperedge structure, the edge sampling procedure generates and encodes different hyperedges in memory. According to the sampling counts for an event, the encoded memory varies under the structure of a random hypergraph. In this experiment, we observed the change in results according to the different study durations.

**Figure 12:**
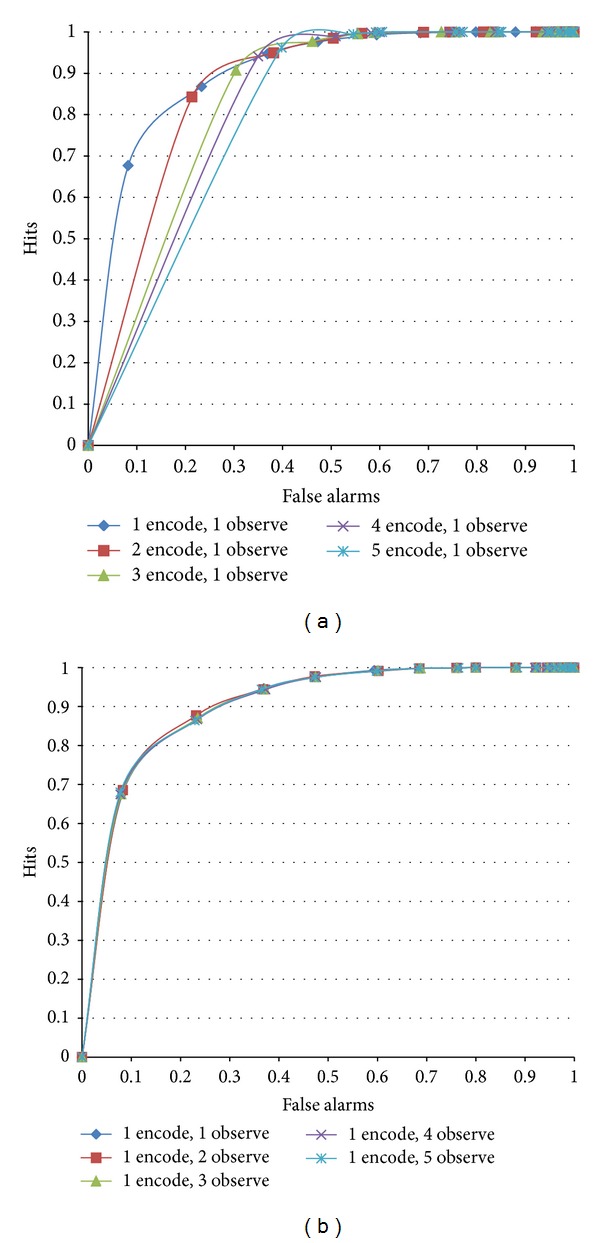
ROC curves for the study duration in random-order edges of (2, 3): repeated (a) encodings and (b) observations.

We repeatedly encoded the same instances at a certain edge configuration with a random order. As an optimal edge condition, the edge orders varied from 2 to 3. The familiarity judgment performance changed according to the number of encodings, as shown in Figure 12(a). When a single encoding was applied, the shape of the ROC curves was symmetric. However, repeated encoding made the memory reveal large false alarms. Five memory encodings showed a similar curve as memory with a low fixed-edge configuration (see Figure 9(a)).

On the other hand, repeated observations were applied to investigate the familiarity judgment performance. If an input instance is judged as old, the instance is repeatedly judged again until the assigned count. In this experiment, we set the count to five. If the input instance is old, the judgment will always be old regardless of the number. However, a new instance can be judged as new and not old, through a random-edge configuration. The study duration can judge exactly whether the input data are old or new by several observations using memory. We predicted that repeated observations would enable false alarms to be corrected.  Figure 12(b) shows the resulting ROC curves. Although the number of false alarms decreased by the repeated number of observations, the shapes of the ROC curves were almost the same.

**Figure 13:**
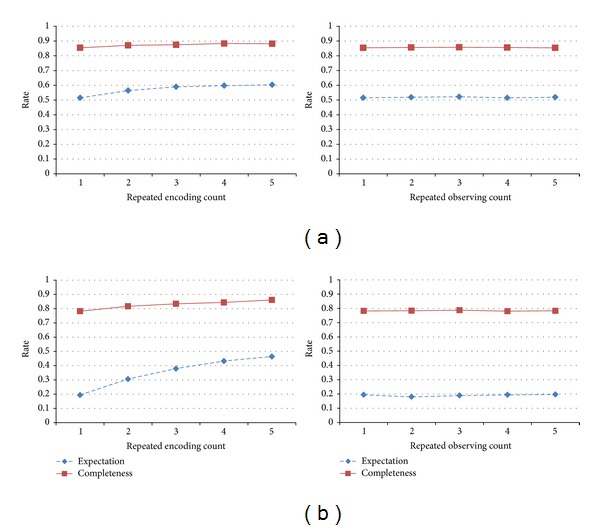
Pattern completion performance based on the study duration. (a)  shows the changes in completeness and expectation for a random-order edge configuration of (2, 3). (b) shows these changes for a random-order edge configuration of (2, 6).

The pattern completion performance was also influenced by the study duration. To evaluate the effect, repeated encodings and observations were applied to the memory process.  Figure 13 shows four results from the different edge configurations. Overall, the expectation and completeness increased according to the number of encodings. In contrast, repeated observations had no effect on the pattern completion performance. Repeated encodings allow the pattern completion performance to increase. With a random-order edge configuration, each hyperedge sample will have a different combination of values. Hence, repeated encodings make the memory model richer and have a high connectivity. In the evaluation of the relationship between the connectivity and performance, the repeated encodings influenced the connectivity. However, repeated observations do not have a relationship with the connectivity, and thus the performance showed no change.

#### 4.3.3. Performance of Context Expectation

As a role of pattern completion, the recognition memory can be used to expect the next context in experience event stream. When a partial input enters, the memory completes missing values via the memory connectivity and generates the complete output. In this experiment, we compare the effect of online incremental learning of hypernetworks. Furthermore, the expectation performance of conventional probabilistic model, Bayesian networks, is compared.

To keep up the event stream in the model, the model needs to encode all of previous data. If a model is intractable to update the new data in real time, the model has to judge and infer based on the old model. As an offline incremental hypernetwork, we set updating sections for every 1000 instances. After building a model *H*_1_^*off*⁡^ which is an offline hypernetwork encoded 1000 instances, the next 1000 instances are judged through the *H*_1_^*off*⁡^. The tested 1000 instances are updated to *H*_1_^*off*⁡^ so that a new model *H*_2_^*off*⁡^ is constructed. With this updating approach, the offline incremental hypernetwork is evaluated to calculate the performance of context expectation. As a controlled model, online incremental hypernetworks is compared. The model updates every instance after judging the new input data.

**Figure 14:**
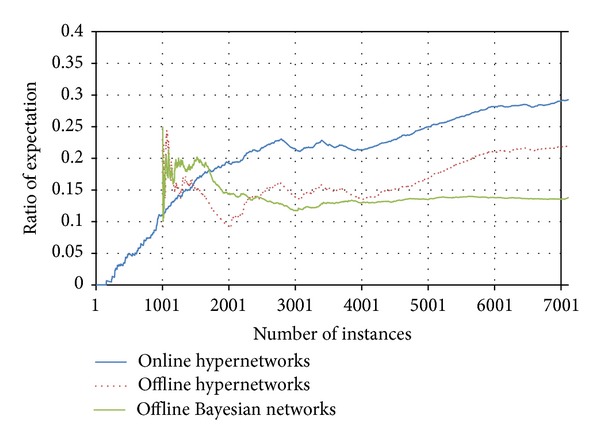
The ratio of expectation performance among online (blue solid line) hypernetworks, offline (red dot line) hypernetworks, and Bayesian networks (green solid line).

In the experiment, three attributes are randomly selected to be a missing value. Then, the remained partial data are used as a cue to complete the missing parts via the encoded recognition memory.  Figure 14 shows the change of the total ratio of context expectation. The blue solid line shows the trend of expectation ratio of incremental memory model along with the updated instances. In the graph, there is an interesting part around 3000th instance, where the ratio decreases. It is caused by the new values in several attributes. If new values in an attribute appear in the event instance, the memory cannot expect the data because there is no same value in the memory. We figure out this trend from Figures 6 and 7 related to the data characteristics. The red dot line that represents offline recognition memory shows a lower performance. The final performances were 29% for online model and 21% for offline model.

Additionally, in order to compare the performance of pattern completion of the recognition memory model, we designed an experiment which represents an expectation of probabilistic model in lifelong experience. We selected Bayesian networks (BN) to infer the missing part from the partial data. If the contextual data is built with probabilistic distribution table, the model can expect the related event from partial instance. The Bayesian network was also tested using the Reality Mining data. To infer an event through the BN model, structure learning and parameter learning are required. Each value in the data is composed of categorical data, so that we used an algorithm from Auton Lab [47]. The parameter learning was executed by using commercial BN product.

Similar to offline hypernetworks, every 1000 instances are used to update the BN model. Then the next 1000 instances are tested whether the model expects the missing values well. Hence, the expectation starts at 1000th instance. At first, the expectation was higher than other memory models. However, the performance decreases by time and the final performance was 13%. The probabilistic model is hard to keep the less probable events. The probabilistic distribution table extracts the most probable values from the conditional probability. This experiment shows that that online hypernetwork is more adaptable than the probabilistic approaches for pattern completion and expectation in lifelong experience.

## 5. Discussion

### 5.1. Tradeoff in Performance Based on the Connectivity

We evaluated the proposed recognition memory model in terms of familiarity. We investigated two functionalities of recognition memory, old/new judgment as explicit memory, and pattern completion as implicit memory. From the various edge configurations, we found a tradeoff in the two functionalities. For old/new judgment, we searched the optimal conditions for a hypergraph structure that resembles the recognition memory based on human behavior. If the memory model merely acts as a judgment model, the memory model should separate old and new instances perfectly. When we model the memory with a high number of fixed-order edges, we can reach the memory goal. However, old/new judgment is an explicit function of recognition memory and only works for complete input data without missing values. Additionally, we focused on another characteristic of recognition memory, that is, the implicit function. When partial data with missing values are assigned as an input value to the encoded memory, the performance is not indicated by the ROC curves, which deals with true and false positives, but by the possibility to generate the original complete data. We found that the explicit and implicit functions have a tradeoff relationship, and thus we need to select the optimal conditions for those two distinguishable processes.

The main criterion for the performance was network connectivity in the memory model. The memory model revealed a different connectivity according to the edge configuration. A model with a large number of fixed-order edges has a tradeoff relationship with a model with a small number of fixed-order edges. The memory model with high-order edges performs well in terms of familiarity judgment but is weak for pattern completion because it constructs a low connectivity. Therefore, when diverse types of edge orders are mixed into the memory model, this tradeoff relationship can be resolved.

### 5.2. Meaning in Lifelong Learning

The proposed model was developed to imitate the functionality of the human brain. During lifelong experience, humans occasionally become confused whether their current situation is familiar and may recognize new situations as old. This phenomenon happens when the person has already experienced a subset of the partial context. Since our hypergraph-based memory model is also constructed by aggregating the subsets of the context, it shows a similar effect in the familiarity judgment.

The purpose of recognition memory of lifelong experience is to recall and predict the user experience based on the previously encoded memory. The role of the recognition memory is determined according to the properties of the input data. When complete data are entered, the memory judges whether the event is old or new. For this function, the memory should perform the recall task well. On the other hand, partial data requires a different procedure based on the recognition memory. The partial data are generated into complete data through the encoded memory, and the completed data contain various combinations including the exact original data. In terms of prediction, our recognition memory model suggests possible data from a partial input. It is assumed that the memory has experienced the possible data before. Similar to memory with false alarms, humans can also be confused regarding their experiences. Furthermore, the new property of our computational model, that is, incremental recognition memory, can explain many unresolved phenomena in human behaviors.

### 5.3. Comparison with Other Models

In order to build a computational recognition memory, previous researches on global matching algorithms [31] have also shown the human-like ROC performance on familiarity judgment. In comparison with the previous models, our proposed hypergraph-based memory model solves new issues related to recognition memory. First, the memory model is tractable to encode categorical data. The recognition memory is highly related to episodic memory and the dominant values of episodic memory are a sort of categorical data. Hypernetworks encode the input data itself into the memory with special connection so that it can include any type of values as they are. Second, our model enables incremental learning without requiring the previously encoded data. In contrary, the global matching algorithms ignored the incremental learning issue and the structure of the models is fixed. Hence, if the model needs to be updated, it has to rebuild itself from all data. Third issue is that our memory model has high memory capacity. In lifelong experience, the detail values of contextual attributes are unlimited. According to human behaviors, new values appear. The recognition memory model should manage the new values. However, the global matching algorithms are hard to handle these issues because of its inflexible structure. In our experimental data, every instance is composed of categorical values and it is sequentially acquired. New values frequently appear in the event stream. Because of these characteristics of Reality Mining data, it is hard to compare our model with the previous global matching algorithms.

In terms of pattern completion, the process is necessary to use in decision making in lifelong learning. Conventionally, the decision making process has used a probabilistic approach. In our experiment, we evaluated the expectation performance with BN and showed that the proposed model outperforms BN. One of the reasons is the memory model which uses hypergraph, which allows high-order relationship between attributes. While BN connects two attributes, our model combines local values rather than attributes. In order to extract the previous data, the combination of attributes needs to be maintained in the model. Hypernetworks contain the individual connection between attributes and it can judge by activation-based memory mechanism. However, BN accumulates all the event relationship to calculate the total probability distribution table and ignores the less probable events. That is why the hypernetworks showed better performance on pattern completion than BN.

### 5.4. Limitation and Applicability

In this model, we assumed that the recognition memory has a crucial role at the early stage of decision making process, similar to that in human beings. However, the proposed memory model covers only low-dimensional categorical data. For application in numerical data, the edge extraction and activation mechanisms should be adapted. For high-dimensional data, a ring-type or line-type network makes a weak correlation between two edges that have a long dimensional distance. Even though high-dimensional data can be encoded, the advantage of a hypergraph structure is ambiguous. For only lifelong experience modeling, a preprocessing step is necessary to categorize signals that can be acquired by the sensing devices. The contextual and behavioral attributes for explaining the experience are recommended to have low dimensionality.

In familiarity judgment, we evaluated the performance using false alarms and hits. The opposite of a false alarm is a false negative case, in which an old event is recognized as new. Through the hypergraph structure, if the memory model uses an activation-based mechanism, the model allows no false-negative cases. To generate false-negative cases in this memory model, the edges and links that are created by the input data should be deleted. In lifelong experience, this is understood as a situation of memory decay, that is, forgetting or aging. If some edges and links that are rarely activated by the next instances are removed, memory decay may be implemented in this memory model. However, we do not deal with the issue of memory decay herein. Therefore, the overall performance of a familiarity judgment in this model is better than that of actual humans.

Another limitation of the proposed model is that it excludes the recollection function. Based on the dual process theory, we postulated that the process for familiarity is different from that for recollection. We assumed that familiarity operates in each single domain and that recollection requires at least two different domains. To implement a recollection, two familiarity memories with different data types are required. Accordingly, the memories are associated with lifelong experiences.

As future work, we need to expand the familiarity memory to recollection memory. Different sensory data such as images and sounds are candidates for recollection memory. After preprocessing to reduce the number of dimensions, multivariate data can be encoded into the memory so that it has the same role as recognition memory, that is, familiarity judgment and pattern completion. Translation between languages and visual information are other possible domains. For language memory, we will attempt word learning. By using the flexible structure of hypergraphs, those various kinds of data can be modeled and be used as a general recognition memory.

## 6. Conclusion

In this paper, we introduced the mechanisms of a hypergraph-based recognition memory model and described the characteristics and considerations of the model for adaption to the functionalities of recognition memory. For memory encoding, we focused on incremental learning and constructing a high-order relationship between nodes. A hypergraph-based model can apply these considerations. From the proposed memory model, we investigated the optimal conditions of the structure to mimic the behavioral performance of humans. When memory assigns random-order edges, the ROC curves for a familiarity judgment show symmetric curvilinear shapes most similar to humans. Furthermore, the memory model was validated to achieve a regular performance even for temporal encoding and the study duration for lifelong learning.

Our model showed a tradeoff in performance with recognition memory because of the connectivity level of the memory structure. A high achievement in familiarity judgment requires a low connectivity, while pattern completion shows a better performance at a high connectivity. The order sizes of the hyperedges showed the opposite correlation with the connectivity. According to the data domain and purpose of the memory model, the connectivity can be manipulated by modulating the model parameters. Based on this computational model on recognition memory, we will try to expand the memory model to enable recollection and apply it to other multimedia domains. We presume that the main problem in accomplishing this will be related to the way the hypergraph structure, which contains the temporal and spatial information, is built.

## Figures and Tables

**Table 1:** Attributes and values of applied Reality Mining data.

Attributes	Number of value categories	Value examples
Time	24	Hours (from 0 to 23)
Location (cell tower ID)	Over 30	Cell tower IDs
Place	4	Home, work, elsewhere, no signal
Phone application	Over 20	Phone, logs, menu, screensaver, and so forth
Contact ID	Over 40	IDs in phone address book
Call direction	2	Outgoing, incoming
Call type	3	Voice call, short message, packet data
Duration	10	Categorized duration in seconds
